# Glucocorticoids after birth trauma and the associated risk of developing posttraumatic stress disorder: a non-randomized open-label pilot trial

**DOI:** 10.3389/fgwh.2025.1557552

**Published:** 2026-01-09

**Authors:** Joanna A. Kountanis, Maria Muzik, Graciela Mentz, Xinyi Zhao, Phillip E. Vlisides

**Affiliations:** 1 Department of Anesthesiology, University of Michigan Medical School, Ann Arbor, MI, United States; 2 Department of Obstetrics & Gynecology, University of Michigan Medical School, Ann Arbor, MI, United States; 3 Department of Psychiatry, University of Michigan Medical School, Ann Arbor, MI, United States; 4 Center for Consciousness Science, University of Michigan Medical School, Ann Arbor, MI, United States

**Keywords:** glucocorticoids, hydrocortisone, postpartum hemorrhage, mothers/psychology, postpartum period, pregnancy, stress disorders—posttraumatic, obstetric

## Abstract

**Background:**

Postpartum posttraumatic stress disorder (PTSD) occurs commonly in individuals after childbirth and is associated with adverse maternal and neonatal outcomes. The primary objective of this pilot study was to determine the acceptability and feasibility of administering glucocorticoids to reduce posttraumatic stress symptoms after a traumatic birth event.

**Methods:**

This was a single-center, non-randomized, open-label pilot trial conducted at Michigan Medicine. Postpartum patients who screened positive for PTSD DSM-V Criterion A (felt a threat to life or injury to self or neonate) were enrolled. Participants self-selected to either (1) receive hydrocortisone within 12 h of the traumatic event or (2) defer hydrocortisone and remain in an observational control arm. Participants were assessed at the time of enrollment and multiple time points postpartum (days 3, 14, and 42) for posttraumatic stress symptoms using the City Birth Trauma Scale. The analysis compared the distribution of PTSD symptom scores in the intervention and control arms over time via weighted generalized estimating equations.

**Results:**

Study recruitment was highly successful, with 133 of 138 eligible patients (96%) enrolled, and only 9 of 261 approached patients (3%) refused to be screened for study participation. Among participants who chose to receive hydrocortisone, the study drug was administered within 12 h of birth in all cases (20/20, 100%). For the primary clinical outcome, PTSD score, 127 participants were included in the longitudinal analysis; *n* = 19 self-selected to receive the hydrocortisone intervention, and *n* = 108 enrolled in the control arm. In the hydrocortisone arm, PTSD scores significantly increased from baseline to postpartum day 14 [6.29, 95% CI (0.36, 12.22), *p* = 0.038] and from baseline to postpartum day 42 [7.46, 95% CI (0.30, 14.61) *p* = 0.041] when compared with the control's change in PTSD scores from baseline to postpartum day 14 and from baseline to postpartum day 42.

**Conclusions:**

These pilot findings demonstrate the acceptability and feasibility of enrolling obstetric patients into a pharmacologic clinical trial immediately postpartum. In those at high risk for PTSD, glucocorticoids did not decrease, and may have increased, PTSD symptomatology. Future larger and randomized trials are needed to confirm or refute these initial findings.

**Clinical Trial Registration**: https://clinicaltrials.gov/study/NCT04852458, identifier, NCT04852458.

## Background/aims

Emerging evidence has demonstrated alterations in neuroactive steroid levels in those with posttraumatic stress disorder (PTSD) (1, 2). Abnormalities in fear extinction learning are hypothesized to be involved in the development of PTSD, and derangements in the cortisol feedback to glucocorticoid receptors have been implicated in contributing to this deficit (3, 4). Human and animal studies have found a reduction of PTSD symptoms with glucocorticoid injection immediately after a traumatic event (5–10). The proposed mechanism is that cortisol disrupts the consolidation of fearful memories. This hypothesis is further supported by Galatzer-Levy et al. (11), who found that variations in the stress-related gene *FKBP5* in humans and mice were associated with abnormal patterns of extinction learning, resulting in genotypes with risk for high fear load. In this study, mice treated with high-dose dexamethasone after traumatic events were found to have temporary alterations in *FKBP5* mRNA in their amygdala; however, the reduction in fear response to a learned cue was permanent. This suggests that glucocorticoid treatment may be effective as an early intervention to prevent PTSD if given while trauma memories are first being formed. Randomized controlled trials administering glucocorticoids to patients in the emergency department and intensive care unit, patients during cardiac surgery, or healthy volunteers after witnessing a traumatic movie have demonstrated a reduction of PTSD symptoms (5–10). A Cochrane review combining the data found moderate evidence to support the use of glucocorticoids in preventing PTSD; however, no studies included obstetric patients (12).

Although not routinely screened for, postpartum PTSD may occur quite commonly in birthing patients. A prospective observational study showed that 20% of individuals who required emergent operative management of postpartum hemorrhage screened positive for PTSD within three months (13). Furthermore, a meta-analysis revealed that 10% of individuals with cesarean deliveries also suffered from postpartum PTSD symptoms (14). Suffering from a postpartum mental illness has far-reaching consequences for patients and children. Individuals with PTSD may lack emotional attachment toward their infant, isolate and neglect themselves, suffer from intrusive flashbacks and insomnia, and have comorbid depression or anxiety. Prevention of PTSD is gravely important. Medications are only marginally effective in treating PTSD (15), and many medications utilized to treat PTSD have unpleasant side effects or are known to pass into breast milk, making them an undesirable choice for postpartum individuals. Cognitive therapies can improve PTSD symptoms (16), but such treatment requires frequent visits for extended periods, which places an additional burden on a mother with a newborn. In contrast, administration of glucocorticoids is well tolerated in the peripartum period, and it seems prudent to investigate if a low-risk therapy, such as a single administration of glucocorticoids, could prevent the development of PTSD or other peripartum mood disorders. This intervention may facilitate both research and improved clinical care on this vulnerable and historically difficult to recruit population (17).

The objectives of this study were to identify obstetric patients at high risk of developing postpartum PTSD and enroll them into an open-label pilot study. Participants could choose to receive hydrocortisone within 12 h of childbirth trauma to potentially mitigate the development of PTSD or other related adverse mental health outcomes. The multiple time points of psychiatric screenings provide a granular assessment of patient symptom trajectories and may also provide further insight into the mechanism of hydrocortisone's effects on memory formation. This open pilot trial is the first to assess the acceptability and feasibility of this drug intervention in obstetric patients. It also provides preliminary data to assist in designing a future randomized controlled investigation.

## Research design and methods

### Pilot trial design

This study was a single-center, non-randomized, open-label pilot trial conducted at Michigan Medicine. The study was approved by the University of Michigan Institutional Review Board (HUM00174658), and written informed consent was obtained from all participants prior to enrollment. The trial was registered at https://www.clinicaltrials.gov (NCT04852458, 4/21/2021, Principal Investigator: JK) prior to participant enrollment. Participants were enrolled from 21 May 2021 to 22 September 2022.

Eligible study participants were obstetric patients delivering at a single quaternary academic medical center who were immediately postpartum. Patients who had an emergent cesarean delivery or suffered from postpartum hemorrhage requiring resuscitation or operating room/interventional radiology management had their medical records screened for study eligibility criteria. Individuals were approached to enroll in a prospective longitudinal, purely observational study, as well as to self-select to receive the study drug hydrocortisone. An open-label study design was used to assess the acceptability of obstetric patients enrolling in a drug intervention study immediately postpartum, given that this has not been studied previously. We aimed to recruit at least 20 patients, as feasible, to receive hydrocortisone. The target sample size for the observational arm of the study was approximately 100 patients, anticipating 10%–15% lost to follow-up.

The inclusion criteria included age ≥18 years of age, screening positive for PTSD DSM-V Criterion A (felt a threat to life or injury to self or neonate), experiencing a postpartum hemorrhage or emergency cesarean delivery, and owner of a smartphone or email account. The exclusion criteria included active uncontrolled psychological disturbances identified by current psychiatric admission, psychiatric consultation during admission, or need for a hospital-appointed sitter during admission. These criteria were applied due to ethical concerns and the necessity of assessing the patient's ability to consent and participate in the study when severe psychiatric symptoms were present. Other exclusion criteria were age <18 years of age, non-English speakers requiring a translator, current corticosteroid use or corticosteroid use during the study period (including betamethasone for promoting fetal lung maturity), and cognitive impairment identified by medical chart review or patients requiring a legal guardian for medical decision making. Additional exclusion criteria for patients who selected to receive hydrocortisone were self-reported hypersensitivity to hydrocortisone, inability to consent and administer study drug within 12 h of the traumatic event, and weight <45 or ≥120 kg.

### Interventions

Patients who met study participant criteria and who opted into receiving intravenous hydrocortisone had it administered by an anesthesia provider. The desired time frame of the drug administration was within 6 h of the traumatic event. Patients were enrolled in the study and were able to receive the study drug up to 12 h from the traumatic event, given existing evidence for its use within this time frame (5), as well as the potential challenges with feasibly enrolling patients and administering the study drug within 6 h. Individuals in the observational arm included those who were not able to be administered hydrocortisone within 12 h of the traumatic event or who declined hydrocortisone administration. They were used as controls to create a quasi-experimental design (Supplementary Figure S1). Hydrocortisone dosing regimen (90–150 mg intravenous) was weight-based: 90 mg was administered to participants weighing 45–59 kg; 100 mg to participants weighing 60–69 kg; 120 mg to participants weighing 70–89 kg; 140 mg to participants weighing 90–99 kg; and 150 mg to participants weighing 100–120 kg (8). The patients were monitored for allergic reactions, side effects of the study drug, and mental status changes.

Covariates (e.g., clinical risk factors) that may be associated with PTSD symptomatology were collected via a self-reported questionnaire and investigator review of the electronic medical record. Measures collected included age, race, education level, income, history of mental illness or substance use disorder, history of physical or sexual abuse, gestational age, parity, body mass index (BMI), mode of delivery, and anesthetics received. These measures were also used to characterize patients with PTSD and depression. In addition, we report the administration of any dexamethasone given intraoperatively for each patient. At our institution, low-dose intravenous dexamethasone (4 mg) is given for all cesarean deliveries or general anesthetics for the prevention of postoperative nausea and vomiting. Low-dose dexamethasone treatment leads to dissimilarity in the potency of direct central and peripheral glucocorticoid actions, suppressing pituitary–adrenal secretion at the pituitary level without replacing endogenous glucocorticoids in the brain. All data were entered and stored in the Research Electronic Data Capture (REDCap) database, a HIPAA-compliant secure web application.

### Outcomes

While the primary aim of this pilot trial was to explore the feasibility and acceptability of enrolling high-risk obstetric patients into a pharmacologic trial for PTSD prevention, the primary clinical outcome was the mean PTSD screening score from questions 3–24 of the City Birth Trauma Scale (18). This scale is a questionnaire developed to measure birth-related PTSD as reported by postpartum individuals. It corresponds directly with the DSM-V criteria for PTSD but is specific to childbirth. The questions' scores range from 0 to 66 and relate to PTSD symptomatology. A higher score indicates more symptomatology. All participants were longitudinally assessed for PTSD symptoms at four time points: baseline enrollment and 3, 14, and 42 days postpartum. The survey was delivered via smartphone text message or email, with the message containing a link to the survey (Qualtrics SMS).

A secondary clinical outcome was the mean depression screening score via the Edinburgh Postnatal Depression Scale (EPDS) (19). This screening was administered at baseline enrollment and 3, 14, and 42 days postpartum. The scores range from 0 to 30 with 10 total questions. This survey was also sent to the participant's smartphone in the same manner as described above.

Participants also completed a validated five-item survey, namely, the Tool to Measure Patient Assessment of Clinician Compassion (TMPACC) (20), at the time of enrollment given the data to suggest that PTSD symptoms may be mitigated after a traumatic event if a patient perceives having received compassionate medical care (21). An analysis was conducted to assess the potential effect of participants' perception of compassionate care during the traumatic event on subsequent PTSD symptomatology.

The memory subscore of the City Birth Trauma Scale served as an exploratory outcome, given the proposed protective mechanism of hydrocortisone on mitigating memory disruption (11). This exploratory analysis was performed for the City Birth Trauma Scale questions 3, 5, 6, and 7, as these items relate to the patient's memory of the traumatic birth:

Q3. Recurrent unwanted memories of the birth (or parts of the birth) that you can't control
Q5. Flashbacks to the birth and/or reliving the experience
Q6. Getting upset when reminded of the birth
Q7. Feeling tense or anxious when reminded of the birth

Additional exploratory analyses included a subgroup analysis within patients receiving steroids to test if receiving steroids in ≤6 h yielded different results compared with those receiving steroids in <12 h.

## Statistical analysis

Statistical analyses were performed in SAS version 9.4 (SAS Institute, Cary, NC, USA). Initial exploratory data analysis techniques, such as means, standard deviations, medians, and interquartile ranges and frequencies, were used to describe the distribution of PTSD, EPDS, and other outcome measures. In addition, histograms assessed symmetry and normality of the distributions. Extreme values were determined and removed from the analysis as appropriate. Missing patterns and rates were assessed. Missing rates were <5% therefore complete case analysis was deemed unbiased and reported in the Results section.

An analysis comparing confounders between intervention and control groups was conducted to address a potentially unbalanced distribution. The comparison between groups was assessed using absolute standardized differences (ASD). Measures with ASD >0.2 were included in the generalized estimating equations (GEE) model described below, in addition to the already identified risk factors: age, parity, mental health history, and receiving or not receiving intravenous dexamethasone (4 mg) for postoperative nausea and vomiting prophylaxis intraoperatively.

The trial was liable to allocation bias since the trial participants were not randomly allocated to treatment. Propensity score (PS) matching was considered, but the unbalanced distribution of relevant confounders was still present. Instead, we used an alternative approach using inverse probability weights to reduce potential confounding. Studies have shown that using inverse probability weights methods can help mitigate allocation bias. Among the potential inverse probability weights methods, we chose the “overlapping weights” method that minimized the impact of low propensity score estimates. A propensity score model was developed using age, BMI, race/ethnicity, education [categorized as less (0) or more (1) than high school], marital status, and income (1, 0–$25,000; 2, 25,001–$50,000; 3, 50,001–$75,000; 4, 75,001–$100,000; 5, >$100,001). We also included binary indicators of anxiety, depression, history of previous PTSD, parity 1 or more, dexamethasone administration, physical or sexual abuse, substance misuse disorder, delivery type, and hemorrhage. Once propensity scores were estimated, we created overlapping weights as (1-PS) for the intervention group and propensity scores for the control group.

The primary analysis for the PTSD score outcome was conducted using a weighted GEE approach. GEE models are particularly useful for this type of analysis because they can accommodate skewed distributions while considering potential clustering of patients without explicit estimation of random effects. Clustering structure is accounted for in the working correlation matrix chosen for the analysis, and an exchangeable correlation structure was used for the present study. Estimates of the differences between the mean PTSD scores for the control and hydrocortisone groups were calculated.

A secondary analysis was performed via the same GEE strategy with the EPDS score outcome. To test for the moderation effect of the compassion score, we included a group compassion score interaction term in the model for both primary and secondary outcomes.

The final set of confounders added to the multivariable models for primary and secondary analysis was similar to the one used in the propensity score model, with the addition of a group indicator (0 = control, 1 = intervention), time period indicators [baseline (reference) and days 3, 14, and 42], and the group–time period and group–compassion interaction score.

Finally, for analyzing the exploratory outcome, we estimated the mean PTSD score at each time point, for both the treatment and the control group, and performed a *t*-test within each time period for each question.

A formal power analysis was not performed given the pilot nature of this study. However, statistical simulation for GEE models supports that estimates of the fixed effect parameters are unbiased for a sample size of ≥100 (22). Sample sizes of ≥50 in a GEE model will have 90% power or more to estimate parameters and standard errors without bias. A significance level of 0.05 was used throughout the analysis.

## Results

**Figure 1:**
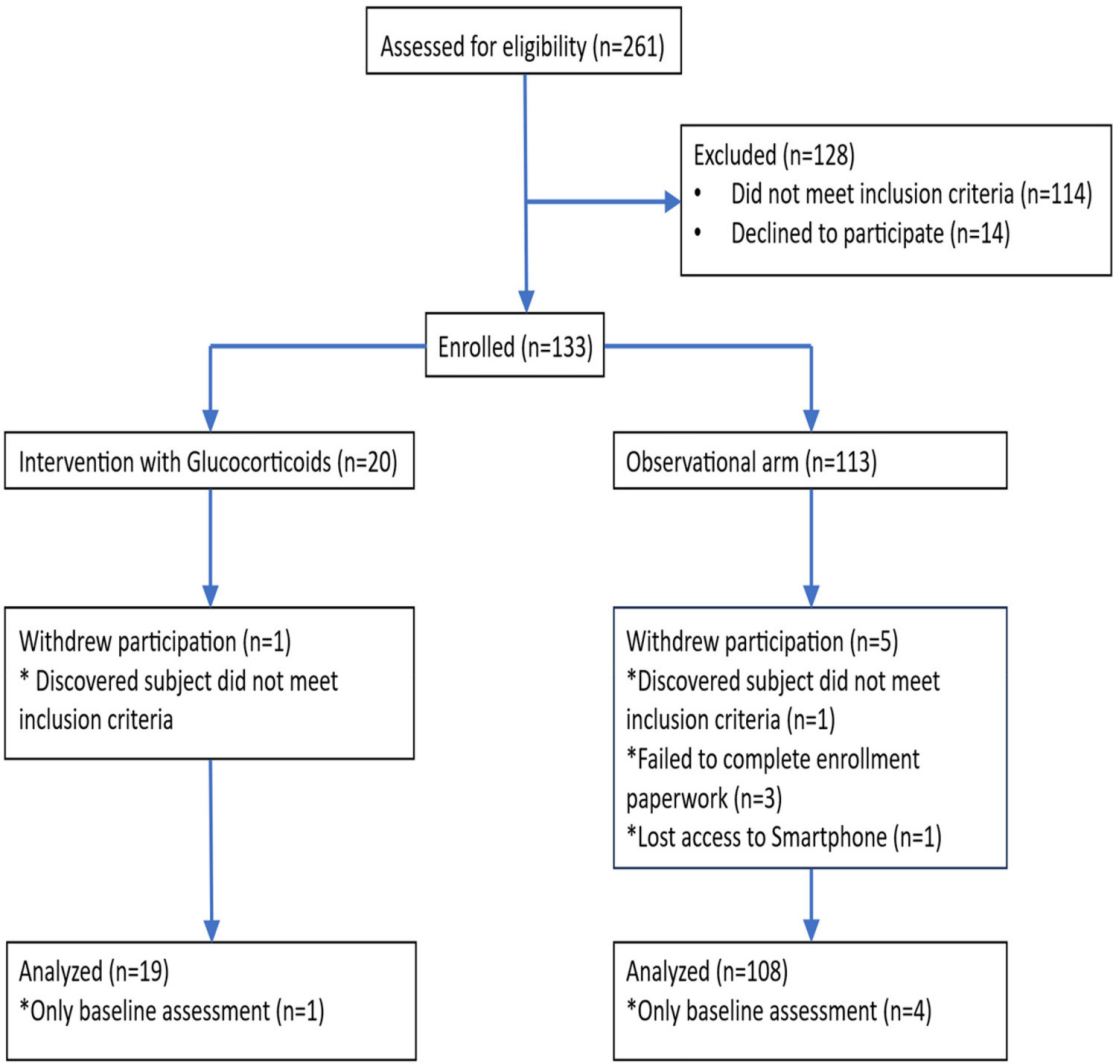
Consort flowchart of participants.

The study flow diagram is presented in Figure 1. In total, 261 patients were approached from 21 May 2021 to 22 September 2022. Of these, 114 patients were deemed ineligible as they did not endorse PTSD DSM-V Criterion A (felt a threat to life or injury to self or neonate) during their emergent cesarean delivery or postpartum hemorrhage as required per eligibility criteria. Nine patients declined to be screened without providing a reason. Five patients were eligible to participate after screening but declined to enroll; three patients were not interested in participating in research, and two patients felt too overwhelmed with a newborn to participate. Finally, 133 patients were enrolled in the study: 20 in the hydrocortisone arm and 113 in the control arm. One patient in the hydrocortisone arm was withdrawn after enrollment, as she was subsequently discovered to meet exclusion criteria based on medical chart review. Five patients were withdrawn from the control arm after enrollment due to different reasons: one lost access to a smartphone, three failed to complete the required enrollment paperwork, and one was found to meet exclusion criteria based on medical chart review. A total of *n* = 127 were analyzed longitudinally, *n* = 108 in the control arm and *n* = 19 in the hydrocortisone arm. There were no reported patient complications in either arm of the study.

Overall, 79.5% (*n* = 101) of participants had a complete data set comprised of a baseline assessment and the three follow-up time points. Loss to follow-up was considered for participants who only had baseline assessments and no follow-up time points. This was 3.9% [*n* = 5 (one steroid, four control)] of participants and did not exhibit a statistically significant different pattern across intervention and control groups.

Demographics of the entire cohort are presented in Table 1. The mean age was 32 years (SD 5.33), approximately 72% who self-identified as non-Hispanic white, 18% who self-identified as non-Hispanic black, and 10% as other. The medical characteristics of the entire cohort included 62% with a history of anxiety, 43% with a history of depression, 5% with a history of bipolar disorder, 14% with a history of non-birth-related PTSD, and 3% with a history of substance use disorder. The birth characteristics of the cohort were that 51% were having their first birth, 62% experienced an emergency cesarean delivery, 56% experienced postpartum hemorrhage, and most patients were awake for their emergency cesarean delivery (78%) or postpartum hemorrhage (87%).

**Table 1:** Patient characteristics (total *n* = 127, control *n* = 108, hydrocortisone *n* = 19).

Characteristics	Full sample	Control	Hydrocortisone	Standardized differences
*N*	127	108	19	
Age, mean (±SD)	31.8 ± 5.33	31.56 ± 5.38	33.21 ± 4.91	0.321
BMI kg/m^2^, median (Q1, Q3)	32.97 (28.38, 38.31)	32.92 (28.43, 38.59)	33.63 (28.42, 37.56)	0.137
Compassion score, median (Q1, Q3)	20 (19, 20)	20 (20, 20)	20 (16, 20)	0.584
Race/ethnicity				
Non-Hispanic Black	23 (18.11%)	18 (16.67%)	5 (26.32%)	0.347
Non-Hispanic White	91 (71.65%)	80 (74.07%)	11 (57.89%)	
Other	13 (10.24%)	10 (9.26%)	3 (15.79%)	
Education				
High school or higher	127 (100%)	108 (100%)	19 (100%)	
Marital				
Single	14 (11.02%)	11 (10.19%)	3 (15.79%)	0.167
With a partner	113 (88.98%)	97 (89.81%)	16 (84.21%)	
Income				
0–$25,000	11 (8.66%)	10 (9.26%)	1 (5.26%)	0.162
$25,001–$100,000	56 (44.09%)	47 (43.52%)	9 (47.37%)	
≥$100,001	58 (45.67%)	49 (45.37%)	9 (47.37%)	
Missing	2 (1.57%)	2 (1.85%)	0 (0%)	
Anxiety				
No	48 (37.8%)	39 (36.11%)	9 (47.37%)	0.23
Yes	79 (62.2%)	69 (63.89%)	10 (52.63%)	
Depression				
No	73 (57.48%)	62 (57.41%)	11 (57.89%)	0.01
Yes	54 (42.52%)	46 (42.59%)	8 (42.11%)	
Bipolar				
No	121 (95.28%)	103 (95.37%)	18 (94.74%)	0.029
Yes	6 (4.72%)	5 (4.63%)	1 (5.26%)	
PTSD				
No	109 (85.83%)	91 (84.26%)	18 (94.74%)	0.347
Yes	18 (14.17%)	17 (15.74%)	1 (5.26%)	
Other psychiatric history				
No	126 (99.21%)	107 (99.07%)	19 (100%)	0.137
Yes	1 (0.79%)	1 (0.93%)	0 (0%)	
Other psychiatric history type				
OCD, ADD/ADHD	1 (0.79%)	1 (0.93%)	0 (0%)	
Missing	126 (99.21%)	107 (99.07%)	19 (100%)	
Physical or sexual abuse				
No	116 (91.34%)	97 (89.81%)	19 (100%)	0.476
Yes	11 (8.66%)	11 (10.19%)	0 (0%)	
Substance misuse				
No	123 (96.85%)	104 (96.3%)	19 (100%)	0.277
Yes	4 (3.15%)	4 (3.7%)	0 (0%)	
Parity				
>1	62 (48.82%)	52 (48.15%)	10 (52.63%)	0.090
1	65 (51.18%)	56 (51.85%)	9 (47.37%)	
Delivery type				
Cesarean	11 (8.66%)	10 (9.26%)	1 (5.26%)	0.437
Emergency cesarean	79 (62.2%)	64 (59.26%)	15 (78.95%)	
Vaginal or assisted vaginal	37 (29.13%)	34 (31.48%)	3 (15.79%)	
Anesthetic type for delivery				
General anesthesia	28 (22.05%)	24 (22.22%)	4 (21.05%)	0.028
Awake	99 (77.95%)	84 (77.78%)	15 (78.95%)	
PONV prophylaxis with dexamethasone				
No	31 (24.41%)	28 (25.93%)	3 (15.79%)	0.251
Yes	96 (75.59%)	80 (74.07%)	16 (84.21%)	
Postpartum hemorrhage				
No	56 (44.09%)	47 (43.52%)	9 (47.37%)	0.077
Yes	71 (55.91%)	61 (56.48%)	10 (52.63%)	
Anesthetic type for hemorrhage				
General anesthesia/deep sedation	17 (13.39%)	15 (13.89%)	2 (10.53%)	0.103
Awake	110 (86.61%)	93 (86.11%)	17 (89.47%)	
Compassion score				
8	1 (0.79%)	0 (0%)	1 (5.26%)	0.813
12	2 (1.57%)	0 (0%)	2 (10.53%)	
13	1 (0.79%)	1 (0.93%)	0 (0%)	
14	2 (1.57%)	2 (1.85%)	0 (0%)	
15	6 (4.72%)	4 (3.7%)	2 (10.53%)	
16	2 (1.57%)	2 (1.85%)	0 (0%)	
17	3 (2.36%)	2 (1.85%)	1 (5.26%)	
18	9 (7.09%)	8 (7.41%)	1 (5.26%)	
19	7 (5.51%)	6 (5.56%)	1 (5.26%)	
20	94 (74.02%)	83 (76.85%)	11 (57.89%)	
Group				
Control	108 (85.04%)	108 (100%)	0 (0%)	
Hydrocortisone	19 (14.96%)	0 (0%)	19 (100%)	

OCD, obsessive compulsive disorder; ADD, attention deficit disorder; ADHD, attention deficit hyperactivity disorder.

**Figure 2:**
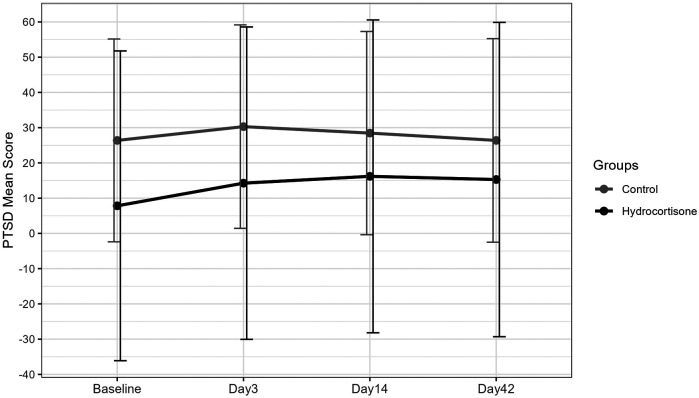
PTSD mean score over time (adjusted by weighted GEE model).

In the multivariable weighted GEE model using inverse probability weights, the compassion score, age, parity, history of anxiety, dexamethasone administration for postoperative nausea and vomiting prophylaxis, and emergency cesarean delivery had statistically significant associations with PTSD scores postpartum (Table 2). For the entire study cohort, PTSD scores had a significant increase on postpartum day 3 [3.91, 95% CI (1.71, 6.11), *p* = 0.001] and postpartum day 14 [2.08, 95% CI (0.3, 3.85), *p* = 0.022]. (Table 2, Figure 2). Mean PTSD scores in the steroid cohort were lower but not statistically significantly different compared with those of the controls at all time points during the study period (Table 2, Figure 2). In the hydrocortisone arm, PTSD scores had a statistically significant increase from baseline to postpartum day 14 [6.29, 95% CI (0.36, 12.22), *p* = 0.038] and from baseline to postpartum day 42 [7.46, 95% CI (0.3, 14.61) *p* = 0.041] when compared with the control's increase in PTSD scores from baseline to postpartum day 14 and from baseline to postpartum day 42 (Table 2, Figure 2).

**Table 2:** Final multivariable weighted GEE regression model with PTSD scores as outcome.

Predictors		Estimate (95% CI)	*P*-value
Intercept		26.38 (−2.4, 55.16)	0.072
Group	Hydrocortisone	−18.55 (−51.77, 14.66)	0.274
	Control	Reference	
Measure time	Day 3	3.91 (1.71, 6.11)	0.001
	Day 14	2.08 (0.3, 3.85)	0.022
	Day 42	0 (−2.38, 2.38)	0.998
	Baseline	Reference	
Group–measure time	Hydrocortisone: Day 3	2.51 (−2.79, 7.82)	0.353
	Hydrocortisone: Day 14	6.29 (0.36, 12.22)	0.038
	Hydrocortisone: Day 42	7.46 (0.3, 14.61)	0.041
	Hydrocortisone: Baseline	Reference	
	Control: Day 3	Reference	
	Control: Day14	Reference	
	Control: Day 42	Reference	
	Control: Baseline	Reference	
Compassion score		−1.85 (−3.33, −0.37)	0.014
Compassion score–group	Compassion score: hydrocortisone	0.98 (−0.71, 2.68)	0.256
	Compassion score: control	Reference	
Age		0.4 (0.15, 0.65)	0.002
Parity	>1	−3.21 (−5.95, −0.46)	0.022
	1	Reference	
Anxiety	Yes	9.53 (4.42, 14.63)	0.000
	No	Reference	
Depression	Yes	2.98 (−1.36, 7.32)	0.178
	No	Reference	
PTSD	Yes	0.36 (−4.14, 4.86)	0.875
	No	Reference	
PONV prophylaxis dexamethasone	Yes	−3.66 (−7.03, −0.28)	0.034
	No	Reference	
Physical or sexual abuse	Yes	4.72 (−2.27, 11.71)	0.185
	No	Reference	
Race, ethnicity	Black, non-Hispanic	−4.83 (−10.15, 0.48)	0.074
	White, non-Hispanic	−0.78 (−5.75, 4.19)	0.758
	Other	Reference	
Substance misuse disorder	Yes	−3.49 (−10.73, 3.75)	0.345
	No	Reference	
Delivery type	Cesarean	5.36 (−1.54, 12.26)	0.128
	Emergency cesarean	5.29 (0.81, 9.77)	0.021
	Vaginal or assisted vaginal	Reference	

Contrast testing of mean PTSD scores within the intervention arm and within the control arm did not demonstrate any statistically significant increase or decrease in PTSD scores over time (Table 3).

**Table 3:** Contrasts testing of mean PTSD scores using overlap weights.

	Estimate (95% CI)	*P*-value
Control		
Day 3–Baseline	3.91 (−25.99, 33.81)	0.798
Day 14–Baseline	2.08 (−27.61, 31.76)	0.891
Day 42–Baseline	0 (−29.99, 30)	1.000
Day 14–Day3	−1.83 (−32.61, 28.94)	0.907
Day 42–Day3	−3.91 (−34.98, 27.16)	0.805
Day 42–Day14	−2.07 (−32.94, 28.79)	0.895
Intervention		
Day3–Baseline	6.43 (−59.43, 72.28)	0.848
Day 14–Baseline	8.36 (−57.6, 74.32)	0.804
Day 42–Baseline	7.46 (−59.48, 74.39)	0.827
Day 14–Day 3	1.94 (−67.67, 71.54)	0.956
Day 42–Day 3	1.03 (−69.49, 71.56)	0.977
Day 42–Day 14	−0.91 (−71.53, 69.72)	0.980

Analysis of the four questions related to memory demonstrated that each question was significantly increased in the intervention group compared with controls at multiple time points postpartum (Table 4). However, the clinical significance of this result is unknown as the difference in means between groups was very small (<1) for each question at each time point (Table 4).

**Table 4:** Analysis of the four questions related to memory using overlap weights.

		Mean		Difference of means	95% CI of mean difference	*P*-value
		Control	Hydrocortisone			
PTSD Q3^a^	Baseline	0.84	1.12	0.28	(−0.1, 0.66)	0.150
	Day 3	1.19	1.66	0.47	(0.14, 0.81)	0.006
	Day 14	0.94	1.54	0.60	(0.19, 1)	0.004
	Day 42	0.63	1.11	0.48	(0.09, 0.87)	0.016
PTSD Q5^b^	Baseline	1.07	1.09	0.02	(−0.39, 0.43)	0.920
	Day 3	1.59	1.74	0.15	(−0.2, 0.49)	0.411
	Day 14	1.19	1.58	0.39	(0.02, 0.77)	0.041
	Day 42	0.67	1.24	0.57	(0.22, 0.92)	0.001
PTSD Q6^c^	Baseline	0.63	0.66	0.03	(−0.33, 0.38)	0.889
	Day 3	0.97	1.63	0.67	(0.25, 1.08)	0.002
	Day 14	0.65	1.33	0.68	(0.24, 1.12)	0.002
	Day 42	0.50	0.94	0.44	(0.05, 0.83)	0.027
PTSD Q7^d^	Baseline	0.81	1.21	0.40	(0.02, 0.78)	0.039
	Day 3	1.02	1.68	0.66	(0.32, 1)	0.000
	Day 14	0.74	1.40	0.66	(0.28, 1.03)	0.001
	Day 42	0.56	1.10	0.53	(0.17, 0.9)	0.004

a PTSD Q3: Recurrent unwanted memories of the birth (or parts of the birth) that you can't control.

b PTSD Q5: Flashbacks to the birth and/or reliving the experience.

c PTSD Q6: Getting upset when reminded of the birth.

d PTSD Q7: Feeling tense or anxious when reminded of the birth.

Multivariable GEE analysis of mean EPDS scores did not demonstrate significant differences for the intervention group compared with controls over time (Supplementary Figure S2, Table S2) Increasing age [0.16, 95% CI (0.01, 0.31), *p* = 0.039], increased parity [−2.84, 95% CI (−4.52, −1.16), *p* = 0.001], history of depression [2.84, 95% CI (0.91, 4.77), *p* = 0.004] and anxiety [3.31, 95% CI (1.59, 5.04), *p* < 0.0001], and history of previous PTSD [4.16, 95% CI (1.27, 7.05), *p* = 0.005] were all significantly associated with postpartum depressive symptoms (Supplementary Table S2).

Exploratory analysis of the cohort receiving steroids in ≤6 h from their trauma yielded comparable results to the primary analysis with the full sample at ≤12 h (Supplementary Tables S3–S5). There were no differences in PTSD scores postpartum over time between the intervention and control arm.

## Discussion

The primary aim of this study was to assess the feasibility and acceptability of enrolling obstetric patients into a pharmacologic trial immediately after birth. Our findings show that postpartum recruitment into an interventional drug study is both practical and well-received, demonstrated by high enrollment rates and timely drug administration within the protocol time window. These results support the ability to implement enrollment during the immediate postpartum period, even following a traumatic birth experience. However, this trial could not demonstrate an association between the administration of hydrocortisone and a reduction of PTSD symptoms in the obstetric population. The intervention group did not show a reduction of PTSD symptoms after steroid administration at any time point within 42 days postpartum compared with controls or their own baseline scores.

This study has many important findings. First, we were able to recruit obstetric patients and maintain their participation in a drug intervention study safely, feasibly, and robustly. Our findings indicate that a history of anxiety is a significant risk factor for developing postpartum PTSD and postpartum depression. Given this association, obstetric providers may consider routine screening for both a history of and current symptoms of perinatal anxiety in addition to standard perinatal depression screening. Recognizing and addressing perinatal anxiety as part of comprehensive perinatal mental healthcare may enable earlier identification of those at heightened psychological risk and support timely intervention to mitigate the development of postpartum PTSD and depression. Finally, the entire study cohort had increasing PTSD scores starting at postpartum day 3 and 14 compared with baseline, which may indicate the first opportunity to identify those at higher risk for developing PTSD.

Our findings also support previous literature describing differing PTSD symptom trajectories for subgroups of patients after birth trauma (23) as well as the significant association of pre-existing psychopathology and postpartum PTSD (13). The significant associations found between PTSD symptoms in those who are having their first child, with an emergency cesarean delivery, with a poor perception of clinicians' compassion, and who have a positive mental health history may further lay the groundwork to identify individuals at high or low risk for developing postpartum PTSD and to create appropriate target preventative and treatment strategies for the patients most likely to benefit.

However, unlike previous randomized control trials, we failed to demonstrate an association between glucocorticoid administration and a reduction of PTSD symptoms. This may be in part due to utilizing a different PTSD screening tool and measuring symptoms at an earlier time period compared with previous studies, which may have precluded our study from finding the same differences in symptoms between cohorts. For instance, Schelling et al. (6) measured patient symptoms at 6 months after an intensive care discharge using a modified Posttraumatic 10 Stress Symptom Inventory. Delahanty et al. (5) measured emergency department patient symptoms at 1 and 3 months post-injury using the Peritraumatic Dissociative Experiences Questionnaire Self-Report Version.

Another explanation of our results may be related to the time of day the hydrocortisone was administered. A recent randomized, placebo-controlled double-blind trial investigated whether a single dose of hydrocortisone given within six hours of trauma would reduce PTSD symptoms at 13 months posttrauma (24). Hydrocortisone was not found to be effective at preventing PTSD compared with placebo. However, they reported a lower prevalence of PTSD in the group who received hydrocortisone if the trauma occurred at night when cortisol levels were low. In this study, all patients received steroids between 8:00 a.m. and 7:00 p.m. due to the research team's work hour constraints.

Pregnancy is considered a state of hypercortisolism, with maternal circulating cortisol levels at delivery influenced by multiple factors, including age, gestational age, BMI, parity, smoking, and psychosocial stressors such as social determinants of health and obstetric violence. Controlling for these factors was beyond the scope of this pilot study, and these confounders may have affected maternal cortisol levels and our study population's response to hydrocortisone administration.

This study had several notable strengths. First, approximately 80% of participants completed the study in its entirety, and only 4% were considered completely lost to follow-up after enrollment, leading to a robust data set for analysis. Our study addressed the current shortcomings present when evaluating patients' postpartum mental health both clinically and within research. As many as 40% of birthing individuals do not attend their postpartum clinical visit (25), and obtaining mental health data via patients' smartphones increased participation and data collection. This led to more accurate longitudinal data reporting and may transform how clinicians and researchers proceed with postpartum patient assessments in the future.

There are several important limitations of this study. First, the study population is limited to only those with smartphone access which limits the generalizability of the results as well as excludes those who may be at high risk for postpartum psychopathology. Due to the narrow 12 h postpartum window for hydrocortisone administration, patients delivering during the weekend days and at night were less likely to be enrolled in the pilot study due to limitations of the research staff's availability to recruit and enroll subjects. Furthermore, the administration of a hydrocortisone dose (90–150 mg intravenously) was adopted from a previously published trial; however, it is possible that the dose may not have been sufficient to reliably disrupt memory consolidation, underscoring the need for a formal dose-finding study to establish the most effective regimen. A limitation of our analysis examining the interventional arm is that very few participants received hydrocortisone alone; of the 19 individuals in the intervention group, only 3 did not receive dexamethasone. This constrained sample size restricts our ability to assess the independent or interactive effects of hydrocortisone and dexamethasone. Because perioperative dexamethasone administration for postoperative nausea and vomiting is common practice, future randomized studies should explicitly control for its use to more accurately isolate the effect of hydrocortisone. Our final limitation is the pilot nature of the study. As a pilot study, we may not precisely detect the treatment effects of hydrocortisone on PTSD symptoms. Although we applied appropriate statistical methods for observational studies to adjust for measured confounding and used weighting techniques to account for potential unmeasured confounding, unknown confounders or imperfect measurement may still influence the results, thereby limiting causal interpretations.

We successfully investigated a high-risk group of individuals who suffered from postpartum hemorrhage or had an emergency cesarean delivery and endorsed a traumatic experience. As a pilot trial, this study was unable to demonstrate a benefit to administering glucocorticoids in reducing PTSD symptoms after a traumatic birth experience. However, this pilot trial provides important data as the necessary first step in exploring this intervention in obstetric patients for future larger randomized investigations.

## Data Availability

The datasets presented in this article are not readily available because the raw/processed data required to reproduce the above findings cannot be shared at this time due to technical/time limitations and as the data also forms part of an ongoing study. Requests to access the datasets should be directed to JK, kountani@med.umich.edu.
