# Supplementary material for: Glucocorticoids after birth trauma and the associated risk of developing posttraumatic stress disorder: a non-randomized open-label pilot trial
Source: Front Glob Womens Health. 2026 Jan 9;6:1557552. doi: 10.3389/fgwh.2025.1557552 (PMC12827617; doi:10.3389/fgwh.2025.1557552)
Supplement: Supplementary file 1 [file Datasheet1.docx]

**Supplemental Fig1. Study Design Flowchart**


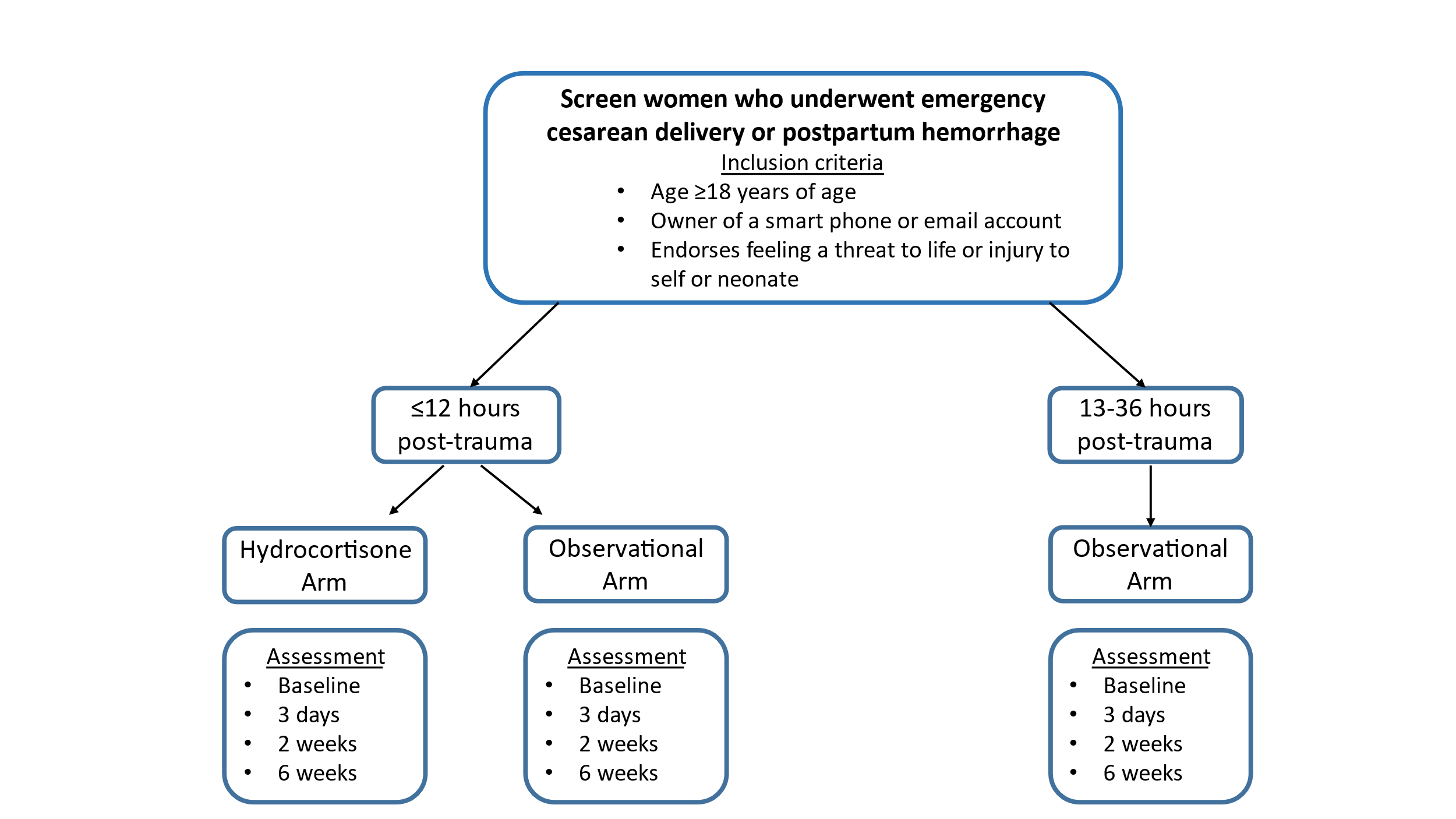


**Supplemental Fig2. EPDS mean score over time (adjusted by weighted GEE model)**


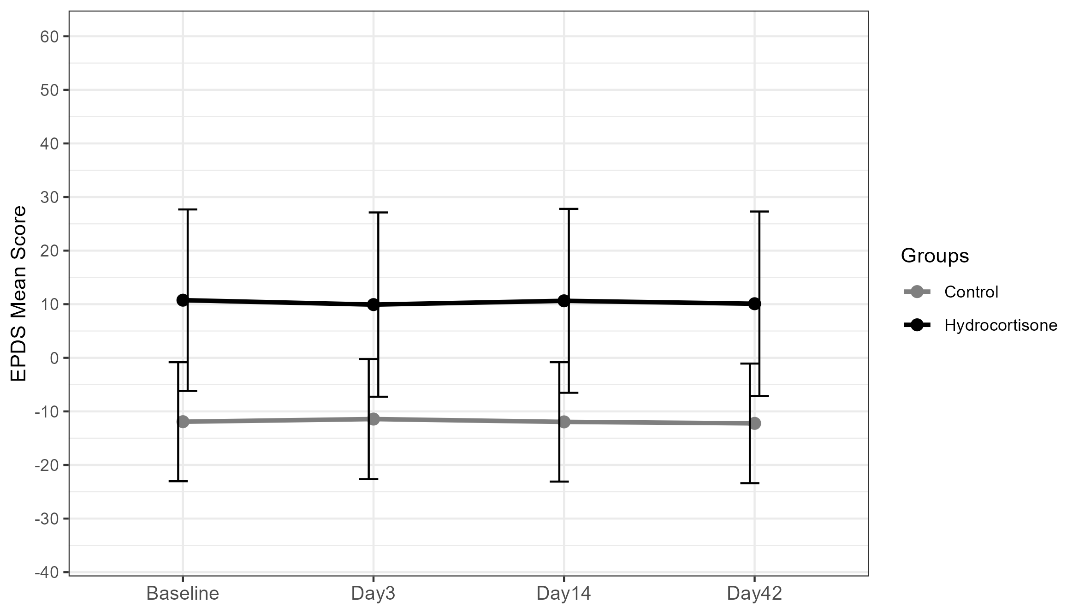


**Supplemental Table 2. Final multi-variable weighted GEE regression model with EPDS Scores as outcome**

| **Predictors** | **Levels** | **Estimate (95% CI)** | **P-value** |
| --- | --- | --- | --- |
| **Intercept** |  | -11.92 (-23.03, -0.82) | 0.035 |
| **Group** | Hydrocortisone | 22.68 (9.9, 35.46) | 0.001 |
|  | Control | Reference |  |
| **Measure Time** | Day3 | 0.49 (-0.93, 1.91) | 0.498 |
|  | Day14 | -0.03 (-0.9, 0.84) | 0.942 |
|  | Day42 | -0.31 (-1.41, 0.79) | 0.582 |
|  | Baseline | Reference |  |
| **Group*Measure Time** | Hydrocortisone:Day3 | -1.32 (-3.96, 1.33) | 0.330 |
|  | Hydrocortisone:Day14 | -0.09 (-2.71, 2.54) | 0.948 |
|  | Hydrocortisone:Day42 | -0.35 (-3.22, 2.52) | 0.809 |
|  | Hydrocortisone:Baseline | Reference |  |
|  | Control:Day3 | Reference |  |
|  | Control:Day14 | Reference |  |
|  | Control:Day42 | Reference |  |
|  | Control:Baseline | Reference |  |
| **Compassion Score** |  | 0.35 (-0.17, 0.87) | 0.190 |
| **Compassion Score*group** | Compassion Score:Hydrocortisone | -1.04 (-1.71, -0.36) | 0.003 |
|  | Compassion Score:Control | Reference |  |
| **Age** |  | 0.16 (0.01, 0.31) | 0.039 |
| **Parity** | 1 | -2.84 (-4.52, -1.16) | 0.001 |
|  | 0 | Reference |  |
| **Anxiety** | 1 | 3.31 (1.59, 5.04) | 0.000 |
|  | 0 | Reference |  |
| **Depression** | 1 | 2.84 (0.91, 4.77) | 0.004 |
|  | 0 | Reference |  |
| **PTSD** | 1 | 4.16 (1.27, 7.05) | 0.005 |
|  | 0 | Reference |  |
| **PONV prophylaxis dexamethasone** | 1 | 1.15 (-0.87, 3.18) | 0.265 |
|  | 0 | Reference |  |
| **Physical or Sexual Abuse** | 1 | 0.89 (-2.26, 4.04) | 0.580 |
|  | 0 | Reference |  |
| **Race_Ethnicity** | Non-Hispanic Black | 2.4 (-0.47, 5.27) | 0.102 |
|  | Non-Hispanic White | 3.34 (0.95, 5.74) | 0.006 |
|  | Other | Reference |  |
| **Substance Misuse** | 1 | -0.93 (-3.39, 1.54) | 0.460 |
|  | 0 | Reference |  |
| **Delivery** | Cesarean | 0.84 (-3.18, 4.86) | 0.683 |
|  | Emergency Cesarean | 0.26 (-1.83, 2.35) | 0.806 |
|  | Vaginal or Assisted Vaginal | Reference |  |

**Supplemental Table 3. Patient Characteristics (intervention <6 hours) (n=117)**

| **Characteristics** | **Full Sample** | **Control** | **Hydrocortisone** | **Standardized Differences** |
| --- | --- | --- | --- | --- |
| n | 117 | 108 | 9 |  |
| Age, mean (±SD) | 31.79 ± 5.35 | 31.56 ± 5.38 | 34.56 ± 4.25 | 0.619 |
| BMI kg/m^2^, median [Q1, Q3] | 32.97 [28.43, 38.51] | 32.92 [28.43, 38.59] | 33.63 [28.5, 37.43] | 0.056 |
| Compassion Score, median [Q1, Q3] | 20 [19, 20] | 20 [20, 20] | 18 [12, 20] | 0.928 |
| Race/Ethnicity |  |  |  |  |
| Non-Hispanic Black | 20 (17.09%) | 18 (16.67%) | 2 (22.22%) | 0.165 |
| Non-Hispanic White | 86 (73.5%) | 80 (74.07%) | 6 (66.67%) |  |
| Other | 11 (9.4%) | 10 (9.26%) | 1 (11.11%) |  |
| Education |  |  |  |  |
| High school or higher | 117 (100%) | 108 (100%) | 9 (100%) |  |
| Marital |  |  |  |  |
| Single | 12 (10.26%) | 11 (10.19%) | 1 (11.11%) | 0.03 |
| With a partner | 105 (89.74%) | 97 (89.81%) | 8 (88.89%) |  |
| Income |  |  |  |  |
| 0-$25,000 | 11 (9.4%) | 10 (9.26%) | 1 (11.11%) | 0.227 |
| $25,001-$100,000 | 50 (42.74%) | 47 (43.52%) | 3 (33.33%) |  |
| ≥ $100,001 | 54 (46.15%) | 49 (45.37%) | 5 (55.56%) |  |
| Missing | 2 (1.71%) | 2 (1.85%) | 0 (0%) |  |
| Anxiety |  |  |  |  |
| No | 45 (38.46%) | 39 (36.11%) | 6 (66.67%) | 0.642 |
| Yes | 72 (61.54%) | 69 (63.89%) | 3 (33.33%) |  |
| Depression |  |  |  |  |
| No | 70 (59.83%) | 62 (57.41%) | 8 (88.89%) | 0.76 |
| Yes | 47 (40.17%) | 46 (42.59%) | 1 (11.11%) |  |
| Bipolar |  |  |  |  |
| No | 112 (95.73%) | 103 (95.37%) | 9 (100%) | 0.312 |
| Yes | 5 (4.27%) | 5 (4.63%) | 0 (0%) |  |
| PTSD |  |  |  |  |
| No | 100 (85.47%) | 91 (84.26%) | 9 (100%) | 0.611 |
| Yes | 17 (14.53%) | 17 (15.74%) | 0 (0%) |  |
| Other psychiatric history |  |  |  |  |
| No | 116 (99.15%) | 107 (99.07%) | 9 (100%) | 0.137 |
| Yes | 1 (0.85%) | 1 (0.93%) | 0 (0%) |  |
| Other psychiatric history type |  |  |  |  |
| OCD, ADD/ADHD | 1 (0.85%) | 1 (0.93%) | 0 (0%) |  |
| Missing | 116 (99.15%) | 107 (99.07%) | 9 (100%) |  |
| Physical or Sexual Abuse |  |  |  |  |
| No | 106 (90.6%) | 97 (89.81%) | 9 (100%) | 0.476 |
| Yes | 11 (9.4%) | 11 (10.19%) | 0 (0%) |  |
| Substance Misuse |  |  |  |  |
| No | 113 (96.58%) | 104 (96.3%) | 9 (100%) | 0.277 |
| Yes | 4 (3.42%) | 4 (3.7%) | 0 (0%) |  |
| Parity |  |  |  |  |
| >1 | 56 (47.86%) | 52 (48.15%) | 4 (44.44%) | 0.074 |
| 1 | 61 (52.14%) | 56 (51.85%) | 5 (55.56%) |  |
| Delivery type |  |  |  |  |
| Cesarean | 10 (8.55%) | 10 (9.26%) | 0 (0%) | 1.173 |
| Emergency Cesarean | 73 (62.39%) | 64 (59.26%) | 9 (100%) |  |
| Vaginal or Assisted Vaginal | 34 (29.06%) | 34 (31.48%) | 0 (0%) |  |
| Anesthetic Type for Delivery |  |  |  |  |
| General Anesthesia | 25 (21.37%) | 24 (22.22%) | 1 (11.11%) | 0.302 |
| Awake | 92 (78.63%) | 84 (77.78%) | 8 (88.89%) |  |
| PONV prophylaxis with dexamethasone |  |  |  |  |
| No | 30 (25.64%) | 28 (25.93%) | 2 (22.22%) | 0.087 |
| Yes | 87 (74.36%) | 80 (74.07%) | 7 (77.78%) |  |
| Postpartum Hemorrhage |  |  |  |  |
| No | 53 (45.3%) | 47 (43.52%) | 6 (66.67%) | 0.479 |
| Yes | 64 (54.7%) | 61 (56.48%) | 3 (33.33%) |  |
| Anesthetic Type for Hemorrhage |  |  |  |  |
| General Anesthesia/Deep Sedation | 15 (12.82%) | 15 (13.89%) | 0 (0%) | 0.568 |
| Awake | 102 (87.18%) | 93 (86.11%) | 9 (100%) |  |
| Compassion Score |  |  |  |  |
| 8 | 1 (0.85%) | 0 (0%) | 1 (11.11%) | 1.436 |
| 12 | 2 (1.71%) | 0 (0%) | 2 (22.22%) |  |
| 13 | 1 (0.85%) | 1 (0.93%) | 0 (0%) |  |
| 14 | 2 (1.71%) | 2 (1.85%) | 0 (0%) |  |
| 15 | 4 (3.42%) | 4 (3.7%) | 0 (0%) |  |
| 16 | 2 (1.71%) | 2 (1.85%) | 0 (0%) |  |
| 17 | 3 (2.56%) | 2 (1.85%) | 1 (11.11%) |  |
| 18 | 9 (7.69%) | 8 (7.41%) | 1 (11.11%) |  |
| 19 | 7 (5.98%) | 6 (5.56%) | 1 (11.11%) |  |
| 20 | 86 (73.5%) | 83 (76.85%) | 3 (33.33%) |  |
| Group |  |  |  |  |
| Control | 108 (92.31%) | 108 (100%) | 0 (0%) |  |
| Hydrocortisone | 9 (7.69%) | 0 (0%) | 9 (100%) |  |

**Supplemental Table 4. Final multi-variable weighted GEE regression model with PTSD Scores as outcome (intervention <6 hours) (n=117)**

| **Predictors** | **Levels** | **Estimate (95% CI)** | **P-value** |
| --- | --- | --- | --- |
| **Intercept** |  | 40.59 (10.88, 70.31) | 0.007 |
| **Group** | Hydrocortisone | -32.32 (-66.91, 2.27) | 0.067 |
|  | Control | Reference |  |
| **Measure Time** | Day3 | 3.9 (1.69, 6.11) | 0.001 |
|  | Day14 | 2.08 (0.3, 3.86) | 0.022 |
|  | Day42 | -0.06 (-2.45, 2.33) | 0.963 |
|  | Baseline | Reference |  |
| **Group*Measure Time** | Hydrocortisone:Day3 | 1.56 (-6.62, 9.74) | 0.709 |
|  | Hydrocortisone:Day14 | 7.01 (-2.11, 16.14) | 0.132 |
|  | Hydrocortisone:Day42 | 8.92 (-2.09, 19.94) | 0.112 |
|  | Hydrocortisone:Baseline | Reference |  |
|  | Control:Day3 | Reference |  |
|  | Control:Day14 | Reference |  |
|  | Control:Day42 | Reference |  |
|  | Control:Baseline | Reference |  |
| **Compassion Score** |  | -2.07 (-3.48, -0.67) | 0.004 |
| **Compassion Score*group** | Compassion Score:Hydrocortisone | 1.65 (-0.17, 3.47) | 0.076 |
|  | Compassion Score:Control | Reference |  |
| **Age** |  | 0.4 (0.15, 0.66) | 0.002 |
| **Parity** | 1 | -3.4 (-6.78, -0.02) | 0.049 |
|  | 0 | Reference |  |
| **Anxiety** | 1 | 9.39 (2.95, 15.84) | 0.004 |
|  | 0 | Reference |  |
| **Depression** | 1 | 1.83 (-3.94, 7.6) | 0.534 |
|  | 0 | Reference |  |
| **PTSD** | 1 | -4.96 (-11.89, 1.97) | 0.161 |
|  | 0 | Reference |  |
| **PONV prophylaxis dexamethasone** | 1 | -5.41 (-8.83, -1.98) | 0.002 |
|  | 0 | Reference |  |
| **Physical or Sexual Abuse** | 1 | 7.33 (0.88, 13.79) | 0.026 |
|  | 0 | Reference |  |
| **Race_Ethnicity** | Non Hispanic_Black | -13.3 (-21.52, -5.09) | 0.002 |
|  | Non Hispanic_White | -9.32 (-16.61, -2.03) | 0.012 |
|  | Other | Reference |  |
| **Substance Misuse** | 1 | -3.32 (-10.14, 3.5) | 0.340 |
|  | 0 | Reference |  |
| **Delivery** | Cesarean | 5.82 (-1.3, 12.95) | 0.109 |
|  | Emergency Cesarean | 6.39 (1.06, 11.72) | 0.019 |
|  | Vaginal or Assisted Vaginal | Reference |  |

**Supplemental Table 5. Weighted analysis of the four questions related to memory (intervention <6 hours) (n=117)**

|  |  | **Mean** | | **Difference of Means** | **95% CI of Mean Difference** | **P-value**^5^ |
| --- | --- | --- | --- | --- | --- | --- |
|  |  | **Control** | **Hydrocortisone** |  |  |  |
| **PTSD Q3**^1^ | Baseline | 0.84 | 0.97 | 0.13 | (-0.42, 0.67) | 0.650 |
|  | Day3 | 1.19 | 1.43 | 0.24 | (-0.16, 0.65) | 0.240 |
|  | Day14 | 0.94 | 1.64 | 0.70 | (0.17, 1.23) | 0.010 |
|  | Day42 | 0.63 | 1.37 | 0.74 | (0.24, 1.23) | 0.003 |
| **PTSD Q5**^2^ | Baseline | 1.07 | 0.96 | -0.11 | (-0.69, 0.48) | 0.714 |
|  | Day3 | 1.59 | 1.63 | 0.04 | (-0.42, 0.49) | 0.880 |
|  | Day14 | 1.19 | 1.35 | 0.16 | (-0.33, 0.64) | 0.533 |
|  | Day42 | 0.67 | 1.04 | 0.36 | (-0.09, 0.82) | 0.119 |
| **PTSD Q6**^3^ | Baseline | 0.63 | 0.86 | 0.23 | (-0.32, 0.79) | 0.413 |
|  | Day3 | 0.97 | 1.64 | 0.67 | (0.05, 1.29) | 0.034 |
|  | Day14 | 0.65 | 1.60 | 0.95 | (0.42, 1.48) | 0.000 |
|  | Day42 | 0.50 | 1.45 | 0.95 | (0.43, 1.47) | 0.000 |
| **PTSD Q7**^4^ | Baseline | 0.81 | 1.01 | 0.20 | (-0.33, 0.72) | 0.465 |
|  | Day3 | 1.02 | 1.67 | 0.65 | (0.16, 1.13) | 0.009 |
|  | Day14 | 0.74 | 1.46 | 0.71 | (0.19, 1.24) | 0.008 |
|  | Day42 | 0.56 | 1.30 | 0.74 | (0.23, 1.25) | 0.005 |

1. PTSD Q3 Recurrent unwanted memories of the birth (or parts of the birth) that you can’t control

2. PTSD Q5 Flashbacks to the birth and/or reliving the experience

3. PTSD Q6 Getting upset when reminded of the birth

4. PTSD Q7 Feeling tense or anxious when reminded of the birth
